# Expert opinion on gray areas in asthma management: A lesson from the innovative project “revolution in asthma” of the Italian thoracic society (AIPO‐ITS)

**DOI:** 10.1002/clt2.70037

**Published:** 2025-02-09

**Authors:** Adriano Vaghi, Raffaele Antonelli Incalzi, Simona Barbaglia, Maria Beatrice Bilò, Francesco Bini, Mauro Carone, Lorenzo Cecchi, Alfredo Antonio Chetta, Andrea Claudio Comel, Fausto De Michele, Giuseppe Insalaco, Antonino Musarra, Giovanni Pomponio, Antonio Spanevello, Silvia Tognella, Alessandro Vatrella, Lina Zuccatosta, Claudio Micheletto

**Affiliations:** ^1^ Former Head of Pneumology and Chief of the Department of Medicine and Rehabilitation Guido Salvini Hospital‐ASST Rhodense Garbagnate Milanese (Milan) Italy; ^2^ Research Unit of Internal Medicine and Geriatrics Department of Medicine and Surgery Fondazione Policlinico Universitario Campus Bio‐Medico Rome Italy; ^3^ President National Patient Association Respiriamo Insieme‐APS Padova Italy; ^4^ Department of Clinical and Molecular Sciences Università Politecnica delle Marche Ancona Italy; ^5^ Allergy Unit Department of Internal Medicine Azienda Ospedaliero‐Universitaria delle Marche Ancona Italy; ^6^ Respiratory Unit ASST Rhodense Garbagnate Milanese (Milan) Italy; ^7^ Division of Respiratory Disease and Respiratory Rehabilitation Istituti Clinici Scientifici Maugeri Pavia IRCCS di Bari Bari Italy; ^8^ Allergy and Clinical Immunology Unit San Giovanni di Dio Hospital Florence Italy; ^9^ Department of Medicine and Surgery University of Parma Parma Italy; ^10^ Pulmonology Unit Pederzoli Hospital Peschiera del Garda (Verona) Italy; ^11^ Respiratory Unit A. Cardarelli Hospital Naples Italy; ^12^ Italian National Research Council (CNR) Institute of Translational Pharmacology (IFT) Palermo Italy; ^13^ Allergy Unit Casa della Salute di Scilla Reggio Calabria Italy; ^14^ Clinica Medica Department of Internal Medicine Azienda Ospedaliero‐Universitaria delle Marche Ancona Italy; ^15^ Istituti Clinici Scientifici Maugeri IRCCS Pulmonary Rehabilitation Unit of Tradate Institute Tradate (Varese) Italy; ^16^ Department of Medicine and Surgery University of Insubria Varese Italy; ^17^ Respiratory Unit Mater Salutis Hospital Legnago (Verona) Italy; ^18^ Department of Medicine Surgery and Dentistry “Scuola Medica Salernitana” University of Salerno Salerno Italy; ^19^ Interventional Pulmonology Unit A. Cardarelli Hospital Naples Italy; ^20^ Respiratory Unit Integrated University Hospital of Verona Verona Italy

**Keywords:** asthma, gray areas, guidelines, real‐life, treatment

## Abstract

**Background:**

Despite the availability of numerous guidelines for asthma management, their recommendations are not consistently implemented in clinical practice. This discrepancy between guidelines and real‐world practice among Italian healthcare professionals was explored during the “Revolution in Asthma” training program, which identified “gray areas” and barriers preventing clinicians from adopting guideline‐based approaches.

**Objective:**

This study aims to analyze the key challenges in asthma management and provide evidence‐based solutions to improve adherence to guidelines in clinical practice.

**Methods:**

A group of experts from the Scientific Committee of the Revolution in Asthma project reviewed the program's findings, focusing on three main areas of asthma management: diagnosis, control, and treatment. The experts summarized clinicians' main needs and questions for each area and provided evidence‐based responses and practical recommendations.

**Results:**

The study highlights critical challenges in asthma treatment, addressing two key questions: (a) What are the possible uses and indications for short‐acting *β*‐agonists in asthma patients? (b) How should asthma treatment be initiated and adjusted based on asthma control? The expert panel developed practical, operational tools to support general practitioners and specialists (pulmonologists and allergists) in optimizing asthma management.

**Conclusion:**

This paper serves as a knowledge co‐creation initiative, bridging the gap between clinical guidelines and daily practice. By offering concrete recommendations, it aims to enhance the application of guideline‐based asthma management among healthcare professionals.

## 1 INTRODUCTION

Despite being one of the most common chronic diseases in pediatric and adult patients,^1^, ^2^ asthma is frequently underdiagnosed^3^ or overdiagnosed.^4^ In addition, a consistent portion of patients is not sufficiently treated or monitored in real‐life.^5^

Over the past 20 years, numerous guidelines have been issued to provide physicians with evidence‐based recommendations on how to diagnose, treat, and monitor patients with asthma^6^, ^7^, ^8^, ^9^, ^10^; however, these recommendations are not always adopted in daily clinical practice.^11^, ^12^

Numerous barriers to the implementation of the guidelines were identified, such as the difficulty of adapting to new knowledge and acquiring new assessment tools, the perception that a one‐size‐fits‐all approach is poorly applicable and irrelevant in real life, and the lack of agreement with the proposed recommendations.^12^, ^13^, ^14^, ^15^ Moreover, guidelines may give different recommendations or suggest diagnostic and treatment pathways that are not entirely overlapping. As a result, physicians are often unfamiliar with the exact content of guidelines and tend to rely more on their own “mind lines”.^16^

The “Revolution in Asthma” training program, conducted in 2021 with the contribution of 400 Italian general practitioners (GPs), pulmonologists and allergologists, aimed to improve knowledge of asthma guidelines (GINA, BTS/SIGN, NICE, NAEPP) among Italian clinicians and to investigate to what extent asthma guidelines are actually followed in daily clinical practice.^17^ The project's novelty was the comparative evaluation of recommendations from different guidelines, an approach that participants appreciated.

The “Revolution project” focused on seven different aspects of asthma management, highlighting, for each one of these aspects, what are the “gray areas” (areas where physicians only moderately agreed with guidelines or areas where, despite a formal agreement, the recommendations were not being implemented), and what are the barriers that prevent clinicians from agreeing or applying guidelines in clinical practice.^17^, ^18^

A group of experts further investigated three of the seven areas included in the Revolution project: asthma diagnosis, control, and treatment. Starting from the real‐life experience derived from the “Revolution project,” the paper further investigates the main needs/questions in each area to offer operational tools to GPs and asthma specialists to improve the management of asthmatic patients in the context of Italian real‐life clinical practice. Therefore, this consensus project qualifies as a sharing of knowledge between the community of physicians working in the field and experts using the tools of evidence‐based medicine and international guidelines.^19^, ^20^, ^21^

## 2 METHODS

### 2.1 Project overview

A group of experts on asthma management (Scientific Committee, SC) composed of 18 members (12 pulmonologists, three allergologists, one internist/geriatrician, one patient association representative, and one methodologist) analyzed the results of the “Revolution project,”^17^ focusing on three aspects of asthma: diagnosis, control, and patient treatment.

Starting from the gray areas detected by the “Revolution project,”^17^ the SC summarized the main needs/questions of clinicians for each area and provided evidence‐based responses and suggestions on how to overcome these needs and answer these questions.

This work represents the conclusion of the training process and the investigation into the real‐life use of guidelines (GLs) initiated with the “Revolution project,” summarizing in this paper the opinion of experts in the treatment area.

#### 2.1.1 The Revolution project

The “Revolution in Asthma” project (www.revolutioninasma.it) aimed to improve both the knowledge and clinical application of asthma management GLs among Italian physicians while also assessing their level of agreement with these guidelines and identifying barriers to their implementation. A total of 400 physicians were involved, including 180 pulmonologists, 100 allergists, and 120 general practitioners (GPs). The project was led by a SC composed of 15 experts (12 pulmonologists, two allergists, and one methodologist) and overseen by a scientific officer.

##### Educational objective

The educational component of the project aimed to improve physicians' understanding and application of asthma GLs by facilitating the comparison of four major guideline frameworks, namely: (1) British Thoracic Society/Scottish Intercollegiate Guidelines Network (BTS/SIGN) 2019^7^; (2) National Institute for Health and Care Excellence (NICE) 2017 and its 2020 update^6^; (3) National Heart, Lung, and Blood Institute—Expert Panel Report (NAEPP‐NHLBI‐EPR) 2007 and its 2020 update^9^; (4) Global Initiative for Asthma (GINA) 2019^22^ with 2020–2021 updates.^10^

The SC prepared a series of educational materials, including the full texts of these GLs (in their original language) and critical insights into their recommendations. This material was organized into seven thematic areas of asthma management: diagnosis, monitoring and control, prevention, pharmacological treatment, severe asthma, acute asthma, asthma in pregnancy/occupational asthma/organization and care delivery.

The SC provided synopses of the GLs in Italian, along with translated excerpts of key sections. The comparative analysis was particularly focused on how these guidelines addressed these seven thematic areas, using both text and tables to highlight differences.

Fifteen online meetings were held to deliver this educational content. The first seven meetings, which were interactive and lasted 2 h each, covered the comparative analysis of the GLs. Each session featured (a) detailed reports on the comparative review of the guidelines, (b) interactive discussions of clinical cases relevant to the guidelines, and (c) thematic insights. These meetings were designed to engage participants actively, allowing for real‐time discussions between the speakers and the participants. All educational materials were also made available to the participants through a digital repository for further consultation.^17^

##### Survey objective

The second objective was to evaluate the physicians' agreement with the GL recommendations and assess their actual application of these recommendations in clinical practice. This was achieved through a structured survey that posed a series of questions across three key dimensions:

1. Level of agreement/disagreement with GL recommendations—This was measured using a 9‐point Likert scale, where a score of 1 indicated “complete disagreement” and a score of 9 indicated “complete agreement.” This scale allowed the SC to quantify the physicians' alignment with specific guideline recommendations.
2. Participants' opinions on clinical issues—Physicians were asked to provide their insights into specific clinical scenarios raised by the GL recommendations. These responses were intended to reflect the participants' operational decisions in real‐world practice, serving as a form of ethnographic data on how physicians mentally structure the GLs in relation to their own clinical experiences.^16^
3. Actual clinical practices—Physicians were queried about their real‐world clinical behaviors, specifically how they managed cases related to asthma in the context of their practice settings. This allowed for a comparison between GL recommendations and actual practice.

The SC analyzed the responses across these three dimensions to identify the areas of strong agreement with GL recommendations (Likert scores of 8–9), areas of strong disagreement (Likert scores of 1–4) and gray areas where there was partial or uncertain agreement (Likert scores of 5–7) or where discrepancies were noted between agreement with GL recommendations, participant opinions, and actual clinical practice.

The analysis identified not only areas of high and low concordance but also highlighted discrepancies between theoretical agreement with guidelines and their practical application. Gray areas also included situations where physicians expressed agreement with the GLs and held positive opinions about them but were unable to implement them fully in clinical practice, often due to external factors such as organizational barriers or resource limitations.

##### Follow‐up and final meeting

The responses from the survey were summarized by the SC and critically discussed during a second series of seven follow‐up webinars. These additional meetings allowed for a deeper exploration of the findings and encouraged further discussion among participants regarding the practical challenges of implementing GLs in everyday clinical practice. During these sessions, additional questions were posed, and the SC worked with participants to propose potential strategies for overcoming identified barriers.^23^

The final meeting of the project was dedicated to summarizing the entire process, highlighting key findings related to agreement and disagreement with GL recommendations, and proposing strategies for improving the implementation of asthma guidelines in Italian clinical practice. The project flowchart is reported in Supplementary Figure S1.

#### 2.1.2 Definition of the expert opinion on gray areas in asthma management

The SC working on the present project was largely made up of previous participants (9/15) (Supplementary Figure S1).

Based on the seven thematic areas covered in the Revolution project,^17^ and considering the participants' requests expressed and the topics' relevance in clinical practice, the SC chose to focus on three areas: diagnosis, asthma control, and therapy. For each area, the SC verified the results of the Revolution project by identifying gray areas and barriers according to the previously formulated definition. The present paper summarizes the opinion of experts in the treatment area, while diagnosis and control are only briefly discussed in the Supplementary Material and will be the subject of future papers.

#### 2.1.3 Gray areas and barriers

Participants in the “Revolution project” expressed maximum agreement with the recommendations summarized in Table 1, common to all guidelines:^6^, ^7^, ^8^, ^9^, ^10^, ^24^

**TABLE 1:** Summary of the key points of agreement among project participants with the guideline recommendations regarding the therapeutic management of asthma.

(a) The goal of therapy is to achieve and maintain overall asthma control, thus minimizing current and future risks, using the least amount of medication to reduce the risk of adverse events.^7^, ^8^, ^9^, ^10^
(b) Inhaled corticosteroids (ICS), alone or in combination, are the most effective background therapy (controller drugs) to achieve the overall therapeutic goal and good asthma control.^6^, ^7^, ^8^, ^9^, ^10^, ^24^
(c) In patients who do not achieve asthma control with low doses of ICS, it is preferable to add a long‐acting β₂‐agonist (LABA)^a^ rather than doubling the dose of the ICS.^7^, ^8^, ^9^, ^19^, ^24^

^a^ Only the NICE guidelines (6) recommend the combination with anti‐LT as the first option in case of uncontrolled asthma with ICS rather than LABA. However, the participants disagreed with this option.

Specifically, for the “therapy” area, the SC chose two queries or “topics of greatest interest” among all the questions identified (see above),^13^, ^14^, ^15^ reformulating them as part of an open discussion, as they represented the issues characterized by the greatest uncertainty and the need for more careful and unambiguous definition by participants in the Revolution project:

a) What are the possible uses and indications for SABAs in patients with asthma?

With two sub‐questions: a1) in a patient with mild asthma, should the physician use, as needed only therapy, a combination of ICS + F or SABA? a2) in a patient with mild–moderate to severe asthma, should the physician use SABA as a reliever? What are the other possible indications for the use of SABA in asthma?

b) How to initiate asthma treatment and adjust it to asthma control?

The SC members were divided into two groups and assigned the literature searches and literature analysis of queries (a) and (b).

#### 2.1.4 Literature review

Literature searches were carried out using the databases: Ovid, MEDLINE, and Embase. The two groups proceeded in parallel to the initial drafting of the answers to the queries. They used the literature selected according to methodological criteria and the GLs selected and compared during the Revolution project (BTS/SIGN, NICE, NAEPP),^6^, ^7^, ^8^, ^9^ which did not have substantial updates after 2021, the post‐2021 versions of the GINA document (2022–2023),^25^, ^26^ and the Spanish GLs (GEMA 2023)^16^ not yet published at the time of the implementation of the Revolution project.

After the first draft, the text was revised by the entire SC and supplemented according to the various suggestions of the experts. In the final drafting of the text, special attention was paid to the recommendations of the aforementioned GLs, which received consensus among the participants, and the context in which the physicians to whom the text is addressed operate (first‐ and second‐level outpatient clinics), as well as the experience of the specialists involved in the project and the needs of patients.^11^, ^17^, ^27^

Therefore, the present project was designed as a knowledge co‐creation project involving family physicians, specialists (pulmonologists and allergists), and SC experts.^19^, ^20^

## 3 SCIENTIFIC COMMITTEE SUGGESTIONS

a) What are the possible uses and indications for SABAs in patients with asthma?

### 3.1 What the guidelines say

In 2018, two papers made a breakthrough in our understanding of the treatment of mild asthma.^28^, ^29^

The 2019 recommendation of the British Thoracic Society (BTS) and the 2017 National Institute for Health and Care Excellence (NICE) guidelines to prescribe SABAs as needed in all patients with asthma yielded uncertain responses.^6^, ^7^ Concurrently, the Global Initiative for Asthma (GINA) recommendation to treat mild patients using ICS + F as needed and reserving SABA as needed, always taken with ICS as needed, as a second option.^22^, ^30^, ^31^ There are therefore serious differences between the guidelines that the group examined.

a1) In a patient with mild asthma, should the physician use, as only as needed therapy, a combination of ICS + F or SABA?

The first gray area identified by the SC, when evaluating the difference between the guidelines' recommendations and the participants' responses, is which therapeutic choice is preferable between the use of a fixed ICS/formoterol combination or a SABA, both administered as needed, in patients with a new diagnosis of mild asthma.

In the Revolution project, over 80% of participants agreed with the use of as‐needed ICS/formoterol in mild asthma, as recommended by the GINA document,^22^, ^30^, ^31^ even though 62% of participants considered it appropriate to use SABA as the sole therapy for patients with occasional symptoms, less than 2 times a month, with normal lung function and no history of previous exacerbations.^17^

The NICE guidelines^6^ recommend that adult patients (>17 years old) with mild, infrequent symptoms and normal lung function consider therapy with as‐needed SABA alone, and the Canadian guidelines^32^ include the option of using as‐needed SABA in patients with very mild asthma and no risk of exacerbations. Similarly, the GEMA 5.3 guidelines (Spain)^24^ include the option of using as‐needed SABA (salbutamol or terbutaline) in the first step of treatment (step 1), but exclusively in patients with occasional mild symptoms that occur at most twice a month in the absence of nighttime symptoms. Additionally, these patients must remain asymptomatic between these episodes, have normal and stable lung function, and have had no exacerbations in the previous year, nor should they present risk factors for exacerbations.

A recent meta‐analysis, which includes the SYGMA studies, demonstrated that the use of a low dose of ICS + F as needed reduces the risk of severe exacerbations and emergency department visits or hospitalizations by 65% compared to the use of SABA alone as needed.^33^

Furthermore, as highlighted in the GINA document (2019–2023),^22^, ^25^, ^26^, ^30^, ^31^ the prescription of SABA alone after diagnosis, combined with their immediate efficacy on symptoms, can create the perception that this therapy is “curative” for asthma. This delays the introduction of ICS and increases the risk of poor adherence to their subsequent prescription.

In contrast, the early introduction of ICS in patients with newly diagnosed asthma improves lung function and disease control while reducing the risk of exacerbations.^33^, ^34^, ^35^

Therefore, available evidence clearly supports that, in mild asthma, assessed based on the level of impairment and risk, treatment with ICS/formoterol as needed is strongly recommended compared to as‐needed SABA treatment. The distinction between patients at low or high risk of exacerbation, as suggested by the Canadian guidelines,^32^ especially when dealing with a newly diagnosed patient, is not always straightforward.

The SC also emphasizes that making an objective new diagnosis of asthma in a truly paucisymptomatic patient, and therefore with so‐called intermittent mild asthma (where SABA as‐needed should be prescribed alone), is not always easy in real‐life. This is because the likelihood of detecting positive diagnostic tests, even those with good sensitivity, such as FeNO and AHR, decreases as the patient becomes more clinically stable and minimally symptomatic.^36^, ^37^

Therefore, once an objective diagnosis of asthma is confirmed (i.e., the certainty of the disease), the SC suggests prescribing ICS/formoterol as needed or with a background ICS therapy instead of a SABA for as‐needed therapy in cases of asthma assessed as mild. The general suggestion is that the prescription of an as‐needed SABA should not be made without prescribing an ICS taken regularly.

a2) in a patient with mild‐moderate to severe asthma, should the physician use SABA as a reliever? What are the other possible indications for the use of SABA as needed in asthma?

The SC acknowledged the divergent opinions of physicians on the use of SABAs in patients with asthma, with polarization between those who commonly use SABAs in clinical practice and those who never use them, as they believe SABAs are primarily responsible for asthma deaths, near‐fatal asthma episodes, and severe flare‐ups.

This difference of opinions stems largely from the messiness over the use of SABAs as sole therapy, often used in individuals at risk for uncontrolled asthma, versus the role of SABAs as relievers in patients on ICS or ICS/LABA. The debate highlighted by the Revolution project also reflects the different positions expressed using guidelines on the use of SABAs.^7^, ^9^, ^10^, ^24^, ^38^, ^39^, ^40^

Based on the results of the Revolution project,^17^ the revision of literature evidence and guidelines,^7^, ^9^, ^10^, ^24^ the SC believed SABAs maintain a therapeutic role as reliever drugs in the specific cases, which are summarized in Table 2.

**TABLE 2:** Indications for the use of as‐needed SABA.

SABA as‐needed are indicated:
As reliever therapy in patients treated with ICS or ICS/LABA (including formoterol if not employed as maintenance and reliever therapy, MART) or triple therapy (ICS/LABA/long‐acting muscarinic antagonist, LAMA)^7^, ^8^, ^9^, ^10^, ^24^
In the treatment of acute asthma, especially in children. Immediate and repeated administration of inhaled SABAs by nebulizer or using a metered dose inhaler with a spacer^7^ is suggested as initial treatment in any emergency situation
In the prevention of exercise‐induced asthma, where SABAs represent the easiest‐to‐use drug, also suitable for children^41^
To perform a bronchodilator responsiveness^7^, ^42^
In case of suspected asthma: in patients with uncertain diagnosis, the SC suggested using SABA alone as needed (if the patient's clinical condition permits it) to interfere as little as possible with the results of the functional investigations planned to further confirm the diagnosis. Once the diagnosis is confirmed, the initial treatment can be modified accordingly

A recent meta‐analysis summarizing the outcome of 22 randomized trials and two observational studies conducted over the past 25 years showed that, when used appropriately within prescribed limits as relief therapy, SABAs do not increase the risk of serious adverse events or mortality.^43^

Furthermore, the use of SABA as a reliever medication, in combination with controller therapy, is a useful indicator of poor asthma control^9^, ^44^, ^45^ and represents an important alert factor for both the physician and the patient. In fact, patients classified as uncontrolled in the MASK‐air® study (Mobile Airways Sentinel NetworK for airway diseases), based on measures of patient outcome reports (PROMs), have the highest use of SABA,^46^ and the NAEPP guidelines 2020 revision emphasizes that patients using SABA more than 2 days a week require clinical reassessment.^9^ 95% of participants in the Revolution project agree with these recommendations.

#### 3.1.1 Critical evaluation of clinical studies on the use of SABAs

Physicians are concerned about the risks of SABA abuse by patients. Large observational studies have established that using more than three 200‐dose SABA canisters per year (corresponding to more than 1 inhalation per day) without adequate regular maintenance therapy with ICS is associated with an increased risk of hospitalization and mortality, regardless of asthma severity.^10^, ^47^, ^48^, ^49^

The most impactful studies on using SABAs have been those on SABA Use IN Asthma (SABINA I, II, III).^47^, ^50^, ^51^, ^52^ These retrospective observational studies investigated the correlation between SABA use and clinical outcomes, such as asthma‐related hospitalizations or exacerbations. The three studies demonstrated an association, but not a causal relationship, between excessive (more than three canisters per year) SABA use and increased risk of asthma flare‐ups and mortality.^47^, ^50^, ^51^, ^52^ The SABINA studies also showed that patients who frequently use SABAs do not use ICS regularly. Underutilization of ICS in patients who would need them leads to worsening of the symptoms, thus favoring greater use of reliever medications with the risk of unfavorable outcomes.^53^ Conversely, as shown in the GOAL study, patients who regularly use ICS‐containing therapies and have well‐controlled asthma rarely used reliever drugs.^54^

Even in real‐life, a clear relationship has been observed between greater adherence to ICS‐containing therapies and a lower frequency of SABA use. In the international multicenter MASK‐air® study, each additional day per week of ICS‐formoterol and ICS + another LABA use was associated with a 4.1% and 8.2% lower risk, respectively, of weekly SABA use.^55^

In real‐life, misuse of SABAs frequently occurs after stopping background therapy with ICS or ICS/LABA.^56^, ^57^, ^58^, ^59^

A possible alternative is using ICS/formoterol as needed as the sole therapy (although regulatory authorities have not yet approved this indication) or the combination of ICS/formoterol as a reliever and maintenance drug (SMART/MART).^26^

The Apparent study^60^ showed that the preferred treatment strategy in different European and non‐European countries was ICS/LABA with or without SABAs and that 85% of patients using ICS/formoterol were also prescribed SABA, thus not fully benefiting from the potential advantages of MART. In a British study,^61^ SABAs were used in more than 50% of patients taking ICS/formoterol, indirectly demonstrating the need for improved asthma control. These results suggest that delegating asthma self‐management to patients without close physician supervision and an effective patient‐physician relationship may not always be a successful strategy.^60^, ^61^

This observation was shared by physicians participating in the Revolution project, who emphasized the need for an appropriate educational pathway to make the MART strategy more effective.^17^ In fact, to achieve an appropriate use of SABAs as reliever therapy, it is necessary to increase patient education and literacy and implement educational programs aimed at improving the symptom interpretation understanding of asthma as a chronic inflammatory disease that requires persistent maintenance therapy with ICS administered with different strategies, and implementation of written action plans.^62^, ^63^ To ensure the effectiveness over time of the instructions initially given to the patient, the SC suggested that certain key concepts should be reiterated at each visit, especially by the GP, and also outside asthma‐specific follow‐up visits. These instructions include the proper use of SABAs as relievers,^53^ the frequency of reliever medication use (SABA or ICS/formoterol), and the notion that the disappearance of symptoms is not equivalent to recovery from the disease. These minimal tips (minimal advice) allow positive messages to be repeated with each visit with minimal time investment.^36^, ^64^, ^65^

b) How to initiate asthma treatment and adjust it to asthma control?

**FIGURE 1:**
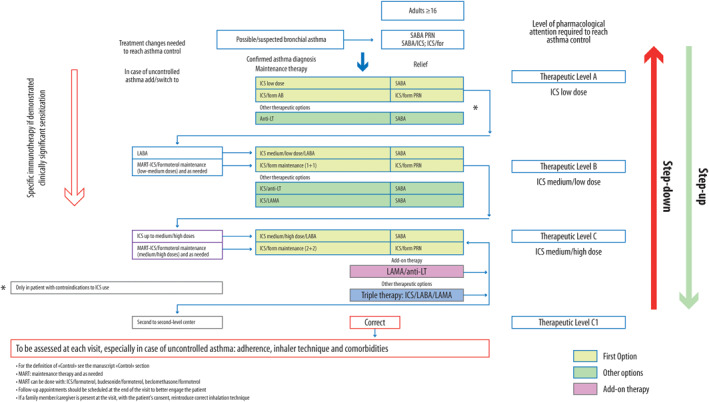
Treatment algorithm for patients with asthma.

Participants in the Revolution project highlighted the need for a treatment algorithm that summarizes the essential patient management steps. The scheme proposed by the SC (Figure 1) is not intended to replace what is recommended by current guidelines but to adapt the guidelines' message to the real‐life Italian context, in a precision‐medicine context, considering the gray areas and barriers identified.

Table 3 summarizes the main elements to consider for proper therapeutic management of the patient according to the SC, which received positive consensus from the participants in the Revolution project.

**TABLE 3:** Summary of the main elements to consider for proper therapeutic management of the patient according to the SC, which received positive consensus from the participants in the Revolution project.

‐ Drug therapy is one of the four cornerstones of patient management, along with monitoring asthma control, education, and reducing the impact of environmental factors and comorbidities. These four fundamental processes are closely integrated with each other^8^, ^9^;
‐ Loss of control should not automatically prompt changes in therapy but rather requires prior assessment of treatment adherence, proper inhaler use, and reduction of exacerbating factors. This could include specific interventions to mitigate indoor allergens, such as a multifactorial approach to allergen‐specific mitigation and management of comorbidities^7^, ^8^, ^9^, ^10^;
‐ The patient should actively participate in the treatment process (shared decision‐making)^61^ and treatment choices and possible alternatives must be shared with the patient based on the agreed goals. Patient involvement and ability to self‐assess and self‐manage can be further enhanced if patients receive a written action plan; ^66^
‐ When assessing incremental response to ICS, it is necessary to consider the complexity of asthma and the existence of different phenotypes (even in mild asthma) to tailor the therapy to patients' needs. Therefore, it is important to identify the patient's treatable traits as early as possible, such as the type of inflammation, the presence of fixed or variable bronchial obstruction, allergic sensitization or intolerance to nonsteroidal anti‐inflammatory drugs^67^, ^68^;
‐ ICS are effective in patients with T2‐type inflammation but less in patients with non‐T2 and paucigranulocytic inflammation.^69^, ^70^, ^71^, ^72^ Additional therapies (add‐ons) with LABA and LAMA or anti‐leukotrienes should be carefully evaluated before deciding on further ICS dosage augmentation.
‐ Specific immunotherapies may also improve the clinical outcome in allergic patients with mild‐to‐moderate asthma.^73^
‐ Some patients with severe asthma, even with very high T2 expression, are characterized by cortico‐resistance and can reach asthma control only through biologic drugs and not by increasing ICS or adding OCS.^74^

In order to immediately visualize the need for the pharmacological intake required by the patient to maintain good asthma control, three levels of pharmacological treatment are presented in the algorithm (Figure 1): low doses of ICS (A), low/medium doses of ICS (B), and medium/high doses of ICS (C). The three levels are defined by the dose of ICS, as this is the most effective class of controller drugs; however, ICS dosages must be carefully weighted for the risk of under‐ or over‐treatment (see Table 4 for daily ICS doses).^6^, ^7^, ^8^, ^9^, ^75^, ^76^, ^77^

**TABLE 4:** Inhaled corticosteroids recommended daily dosages in adults and adolescents over 12 years of age.

ICS (alone or in combination with LABA)	Total daily ICS dose (μg)^a^		
	Low	Medium	High
Beclometasone dipropionate (pMDI, standard particle, HFA)	200–500	>500–1000	>1000
Beclometasone dipropionate (DPI or pMDI, extrafine particle, HFA)	100–200	>200–400	>400
Budesonide (DPI or pMDI, standard particle, HFA)	200–400	>400–800	>800
Ciclesonide (pMDI, extrafine particle, HFA)	80–160	>160–320	>320
Fluticasone furoate (DPI)	100		200
Fluticasone propionate (DPI)	100–250	>250–500	>500
Fluticasone propionate (pMDI, standard particle, HFA)	100–250	>250–500	>500
Mometasone propionate (DPI)	Depends on the DPI device—see product information		
Mometasone propionate (pMDI, standard particle, HFA)	200–400		>400

Abbreviations: DPI, dry powder inhalers; HFA, hydrofluoroalkane; ICS, inhaled corticosteroid; LABA, long‐acting beta‐agonist; pMDI, pressurized metered‐dose inhaler.

^a^ Daily doses are shown as metered doses. Please refer to Product Information for delivered doses.

*Source*: Global Initiative for Asthma (GINA 2022).^25^

Regarding the therapeutic algorithm to be adopted among the participants in the Revolution project, one of the recommendations that reached the widest consensus is the proposal of the BTS guidelines, which do not include differentiated therapeutic steps but a modulation of therapy according to the level of control.^7^ Conversely, the participants expressed perplexity about the GINA recommendation to distinguish patients into two tracks because the criterion for selecting patients seemed artificial, and the need for proper patient self‐management was deemed necessary for both tracks.^17^

#### 3.1.2 Therapy initiation in patients with confirmed diagnosis

The initial choice of background therapy depends on the assessment of the level of severity found before initiating therapy (Figure 1).^6^, ^7^, ^8^, ^9^, ^10^

Since the likelihood of an asthma diagnosis is related to the variability of symptoms and respiratory function,^36^ symptomatic patients who receive a diagnosis based on spirometry results are usually characterized by at least mild‐to‐moderate persistent asthma^78^, ^79^, ^80^ requiring maintenance therapy with ICS (fixed‐dose or as needed), as demonstrated in the meta‐analysis by Ni Chroinin et al.^81^ Subjects with newly diagnosed asthma and bronchial obstruction appear to benefit more from initiating ICS/LABA therapy than ICS alone at the same dose in terms of symptom control and respiratory function, but not in reducing the risk of exacerbation; conversely, initiation with higher dosages of ICS is more effective in reducing the risk of exacerbations.^81^

The SC suggests starting with ICS/LABA with moderate/high doses of ICS (MART or ICS/LABA with as‐needed SABA) in patients whose diagnosis was made following a severe exacerbation.

#### 3.1.3 Therapeutic level A: Low doses of ICS

This therapeutic level coincides with step 2 of the GINA (tracks 1 and 2), NAEPP‐EPR3 and Spanish guidelines.^8^, ^9^, ^10^, ^24^

ICS are the reference maintenance treatment for persistent asthma.^6^, ^8^, ^9^, ^10^, ^24^ Regular use of ICS, even at low doses, is associated with a decreased risk of exacerbations, hospitalizations, emergency room visits, or death from asthma.^75^, ^76^, ^77^ Undertreatment or nontreatment of inflammation (also present in mild asthma) with ICS results in worsening asthma control, airway remodeling, and probably a more rapid decline in lung function. The long‐term benefit of daily low‐dose ICS has also been demonstrated in patients with mild asthma and intermittent symptoms present ≤2 days per week.^82^, ^83^

Similarly to what is reported in the Spanish guidelines,^24^ the SC suggested as first‐choice either daily ICS and SABAs as needed or ICS/formoterol as needed (Figure 1), and as second choice antileukotrienes and SABAs as needed reserved for patients with severe contraindications to ICS.^84^

The SYGMA studies compared the efficacy of three regimens in patients with mild persistent asthma: budesonide/formoterol as needed in a single inhaler, budesonide with SABAs as needed, and SABAs as needed.^28^, ^29^ ICS/formoterol as needed reduced the risk of severe exacerbations by 60%–64% compared with SABAs as needed (SYGMA 1–2)^28^, ^29^; moreover, ICS/formoterol as needed was not inferior to continuous treatment with ICS in preventing severe exacerbations but resulted in less symptom control (SYGMA 1–2)^30,31^ and less improvement in respiratory function (SYGMA 2).^29^ Of note, the total ICS dosage taken by patients in the ICS/formoterol as‐needed arm was lower than that in the regular budesonide arm.

The improved control of symptoms and respiratory function observed using ICS as maintenance is likely related to a more consistent suppression of inflammation and a better bronchoprotection (reduction in nonspecific bronchial reactivity) that can be achieved with regular administration. Studies on validated pharmacokinetic/pharmacodynamic models show that bronchoprotection is significantly reduced when ICS is taken irregularly (<50%) or as needed. However, ICS with greater persistence on the glucocorticoid receptor may partially compensate for the irregularity of intake.^85^, ^86^

In the pragmatic open‐label studies Novel START^87^ and PRACTICAL^88^ treatment adherence with budesonide taken continuously was 56%–60% and lower than that measured in the controlled SYGMA 1 and 2 studies, respectively.^53^ Subsequent analysis of Novel START and PRACTICAL showed that the superiority of ICS/formoterol administered as needed in preventing severe flare‐ups decreased as the patient adherence to regular daily ICS improved.^53^

As mentioned in the GINA guidelines,^10^ the use of ICS/formoterol as needed has two advantages over daily ICS use, as it helps avoid SABA misuse and reduce severe exacerbations even at the expense of symptom control. However, some studies^89^, ^90^ have shown a significant relationship between symptom control and flare‐ups. This relationship is less evident in mild asthma than in moderate/severe forms, where symptom frequency is an important predictor of exacerbations. Therefore, appropriate therapy must be implemented to pursue both goals, namely, control of symptoms (impairment) and exacerbations (future risk), particularly in patients with moderate persistent asthma.^91^

In conclusion, according to the SC, the physician should evaluate the advantages and disadvantages of continuous ICS therapy versus as‐needed ICS/formoterol for each individual patient, keeping in mind both the pragmatic advantages of as‐needed administration and the greater degree of symptom control achieved with maintenance ICS, while tailoring the therapeutic approach to patient preferences, expectations, inclinations, and phenotypic characteristics.^92^

#### 3.1.4 Therapeutic level B: Low/medium doses of ICS

This therapeutic level coincides with step 3 of the GINA, NAEPP and Spanish guidelines.^9^, ^10^, ^24^

For patients who do not achieve good control with low daily doses of ICS, alternative treatments include combining them with a LABA or antileukotriene or doubling the dose of ICS.^9^, ^10^, ^24^ The most effective option to improve asthma control appears to be the combination of low‐dose ICS with a LABA (vilanterol, salmeterol, formoterol, indacaterol), administered with either a single (preferred option) or separate inhalers.^93^

A meta‐analysis conducted by Ducharme et al. including 48 studies (15,155 participants, including 1155 children and 14,000 adults), showed that the combination of ICS and LABA is moderately more effective in reducing the risk of exacerbations than a higher dose of ICS. Moreover, continued ICS/LABA therapy results in greater improvement in lung function and symptoms and reduced use of reliever medications compared with a higher dose of ICS.^94^ LABA combination therapy also represents the first choice in patients who do not achieve adequate asthma control with low/medium doses of ICS.^94^

In subjects who do not tolerate LABA, medium‐dose ICS can be used, although this alternative has been shown to be less effective than the combination of ICS with LABA.^94^, ^95^, ^96^

Another option combines low doses of ICS with a leukotriene receptor antagonist, which has proven superior to monotherapy with medium doses of ICS. However, it is not as effective as the combination of ICS and LABA.^97^, ^98^

Both SABA and ICS/formoterol can be used as reliever drugs in combination with ICS/LABA. In patients using ICS/formoterol as needed, the next therapeutic step would preferably be ICS/formoterol at a fixed dose and as needed (MART).^99^, ^100^, ^101^ However, transitioning from ICS/formoterol as needed to MART can be complex, and the criteria for this transition are still under discussion.^63^, ^102^, ^103^ Participants in the Revolution project^17^ were uncertain about the exact frequency of symptoms and number of inhalations per day or week that can be used as a baseline to begin the transition from as‐needed to continued therapy, also considering the wide range of use and number of inhalations per day recommended for each drug.^102^, ^104^

The SC agreed with the proposal of Beasley^103^ and the GINA guidelines^10^, ^25^ to set this baseline at a minimum of seven inhalations of ICS/formoterol per week (once a day or differently distributed within the week). The step‐up strategy should start from two administrations of ICS/formoterol per day (1 + 1) and as needed (low doses of ICS), to two for two administrations (2 + 2) and as needed (medium doses of ICS) (Figure 1).^103^, ^105^ In patients who need less than two doses as needed per week, therapy can be reduced but not discontinued while maintaining the as‐needed drug use. The proposed algorithm will be validated by a prospective study: Anti‐Inflammatory Reliever Algorithm Study.^105^

In line with this study,^104^ the SC emphasized that this algorithm can be implemented in real‐life only if the patient agrees with the treatment choice, is specifically educated on asthma, has a written action plan, and can acquire, over time and with the help and tutoring of the physician, the skills necessary for self‐management of the disease.

The advantages and disadvantages of each therapeutic choice should be considered and discussed with the patients to meet their expectations and their characteristics.^106^ Single daily administration of ICS/LABA is the preferred option for 84% of participants, according to the Revolution project,^17^ as they are easier to manage and give the patient a feeling of immediate improvement of symptoms.^17^, ^107^, ^108^, ^109^ Similarly, the real‐life Salford study showed that daily single administration of fluticasone furoate plus vilanterol allows, compared with other inhaled therapies, rapid achievement of good adherence and better asthma control in a heterogeneous population of asthmatic patients.^110^

In the MASK‐air® study, greater adherence (use of the medication on ≥80% of weekly days) was also observed for ICS + another LABA (75.1%) compared to ICS + formoterol (59.3%), despite both groups showing similar asthma control.^55^

Real‐life experiences also suggest that continuous and as‐needed ICS/formoterol therapy may not always be sufficiently understood and implemented correctly by patients.^60^, ^61^ However, a significant proportion of patients seem to prefer as‐needed therapy for fear of using excessive doses of medication they do not feel necessary.^17^

#### 3.1.5 Therapeutic level C: Medium/high doses of ICS

This therapeutic level coincides with steps 4–5 of the GINA, NAEPP, EPR3 and Spanish guidelines.^8^, ^9^, ^10^, ^24^

In patients with uncontrolled asthma treated with low/medium doses of ICS/LABA, the ICS dosage should be increased after re‐evaluating and correcting possible risk factors, such as adherence to therapy, inhaler use, and comorbidities.^9^, ^10^, ^24^, ^81^, ^111^, ^112^ Patients under MART can double the dosage to two inhalations twice daily and as needed. In special situations, daily inhalations can also be increased to three‐to four‐times a day or even eight inhalations per day in patients who tolerate high doses of LABA.^67^

Literature data show that the medium‐dose MART strategy led to a reduction in severe exacerbations compared with fixed‐dose treatment and SABA, although the dose of budesonide used with the MART strategy was higher than with the fixed‐dose strategy (943.5 vs. 684.3 μg/day).^68^

In patients treated with fluticasone furoate 100 mcg/day or fluticasone dipropionate 500 μg/day therapy in combination with a LABA and with uncontrolled asthma, ICS can be doubled with the advantage of maintaining the same LABA dosage.^6^, ^9^, ^10^, ^24^

The SC suggested increasing the ICS dosage only after assessing patients' treatable traits; in fact, the likelihood of a good response to ICS depends partially on the type of inflammation and is higher in patients with indicators of T2‐type inflammation.^69^, ^70^, ^113^, ^114^, ^115^ In patients with low or inconclusive indicators of T2‐type inflammation, many symptoms, bronchial obstruction, or a history of smoking and taking ICS/LABA with medium‐dose ICS, it is best to consider adding a LAMA or an antileukotriene^116^, ^117^ or with an antileukotriene (in atopic patients with symptoms of rhinitis),^118^ before increasing the dose of ICS.

In patients with uncontrolled asthma treated with medium/high doses of ICS, the SC suggested adding a LAMA (Thiotopium Br) on top of the current therapy to reduce exacerbations and improve symptoms and respiratory function.^116^, ^117^, ^119^ Triple therapy with a single inhaler, “single inhaler triple therapy,” may lead to improved treatment adherence and appears to improve symptoms and respiratory function and reduce exacerbations in patients with uncontrolled asthma treated with medium/high‐dose ICS and LABA.^74^, ^120^

According to the SC, patients who still present uncontrolled asthma despite high doses of ICS/LABA and possibly a LAMA should be referred to a specialized center for evaluation and possible diagnosis of severe asthma. In these patients, increased expression of T2 markers should not induce increased use of ICS but guide toward a biological drug as elevated T2 cytokine levels are associated with corticosteroid resistance.^41^, ^42^

#### 3.1.6 Step down

Stable patients for 3–6 months may follow a step‐down pathway that mimics the step‐up approach in reverse.^9^, ^10^, ^24^ The use of continuous and as‐needed ICS/formoterol allows for rapid step‐up and step‐down since doses can be easily varied, particularly when therapy is co‐managed with an experienced patient.^10^, ^103^, ^105^

Before initiating the step‐down, the SCs suggested checking for known or predictable risk factors for exacerbations (e.g., pollination season in an atopic subject or close to the winter season in a subject with frequent post‐viral exacerbations). A patient who has had at least two exacerbations in the previous year should be considered uncontrolled and maintain the same therapy even if they currently exhibit limited symptoms, especially after recent respiratory infections or allergen exposure.^8^, ^9^ Intermittent seasonal therapy (therapy prescribed during periods of seasonal exposure) may be considered in patients who, in previous years, have shown a loss of asthma control only in the season when they are exposed to sensitizing allergens (pollens or molds).^8^, ^9^ Furthermore, the SC suggested, before the period of maximum allergic exposure, to start daily therapy with low‐dose ICS/LABA. In patients using ICS/formoterol as needed, the suggested step is the transition to the MART strategy to improve the level of bronchoprotection.^85^

Finally, the SC highlighted that drug therapy and specific immunotherapy complement each other to pursue improvement in asthma control and should not be interpreted as exclusive. Even in this case, the therapeutic strategy should be individualized in collaboration with the patient.^8^, ^9^

In the near future, to improve control and the adoption of personalized therapeutic strategies, it is desirable to increasingly use mHealth self‐monitoring tools such as MASK‐air® and the integration of Patient‐Reported Outcome Measures (PROMs) in asthma management.^46^, ^47^, ^48^, ^121^, ^122^, ^123^, ^124^

## 4 CONCLUSION

Starting from the results of the Revolution project, a group of experts in asthma reviewed the main gray areas and needs in asthma treatment in Italian clinical practice, providing operational tools to GPs and asthma specialists for asthma management that are based on a careful evaluation of guidelines and literature evidence and, at the same time, taking into account the context of care, the experience of physicians (SC and participants) and the needs of patients. Specifically, this paper provides suggestions on how and when to initiate treatment with SABAs. It proposes a treatment algorithm that summarizes the essential steps of patient management by adapting the message of the guidelines to the Italian context but hypothesizes that the proposed suggestions are extensible to the broader healthcare context that shares the gray areas and barriers identified.

## AUTHOR CONTRIBUTIONS

**Adriano Vaghi**: Conceptualization; writing—review and editing; writing—original draft; data curation; formal analysis; investigation. **Raffaele Antonelli Incalzi**: Investigation; data curation; formal analysis; writing—review and editing. **Simona Barbaglia**: Investigation; formal analysis; data curation; writing—review and editing. **Maria Beatrice Bilò**: Data curation; formal analysis; investigation; writing—review and editing. **Francesco Bini**: Investigation; formal analysis; data curation; writing—review and editing. **Mauro Carone**: Formal analysis; data curation; investigation; writing—review and editing. **Lorenzo Cecchi**: Investigation; formal analysis; data curation; writing—review and editing. **Alfredo Antonio Chetta**: Investigation; writing—review and editing; formal analysis; data curation. **Andrea Claudio Comel**: Data curation; formal analysis; writing—review and editing; investigation. **Fausto De Michele**: Investigation; writing—review and editing; formal analysis; data curation. **Giuseppe Insalaco**: Investigation; writing—review and editing; formal analysis; data curation. **Antonino Musarra**: Data curation; formal analysis; writing—review and editing; investigation. **Giovanni Pomponio**: Investigation; writing—review and editing; formal analysis; data curation. **Antonio Spanevello**: Data curation; formal analysis; writing—review and editing; investigation. **Silvia Tognella**: Investigation; writing—review and editing; formal analysis; data curation. **Alessandro Vatrella**: Data curation; formal analysis; writing—review and editing; investigation. **Lina Zuccatosta**: Investigation; writing—review and editing; formal analysis; data curation. **Claudio Micheletto**: Investigation; writing—review and editing; formal analysis; data curation.

## CONFLICT OF INTEREST STATEMENT

A. Vaghi received payment or honoraria as a speaker from A. Menarini, AstraZeneca, Chiesi and GlaxoSmithKline. R. Antonelli Incalzi received payment or honoraria as a speaker from Angelini, A. Menarini, Aristea Srl, Fenicia Events and Communications, MCC Srl, METIS and consultation fees from Editamed Srl, Ethos, GlaxoSmithKline, LT3, Medineos, Merck Sharp and Dohme, Moderna, and SanitaNova Srl. He owns shares of Recordati. M.B. Bilò received payment or honoraria as a speaker from AstraZeneca, GlaxoSmithKline, Recordati and Sanofi. M. Carone received payment or honoraria as a speaker from Boehringer Ingelheim and GlaxoSmithKline. L. Cecchi received payment or honoraria as a speaker from ALK, AstraZeneca, GlaxoSmithKline, Novartis, Sanofi and Thermofisher and consultation fees from ALK, A. Menarini, AstraZeneca and Thermofisher. A.A. Chetta received payment or honoraria as a speaker from Chiesi and GlaxoSmithKline and a research grant from AstraZeneca. A.C. Comel received payment or honoraria as a speaker from A. Menarini, AstraZeneca, Chiesi, Fujifilm, GlaxoSmithKline and consultation fees from Boston Scientific. F. De Michele received payment or honoraria as a speaker from Chiesi and GlaxoSmithKline and consultation fees from AstraZeneca. G. Insalaco received payment or honoraria as a speaker from Bioprojet and ResMed and consultation fees from Bioprojet and Axome Therapeutics. A. Musarra received payment or honoraria as a speaker from A. Menarini, AstraZeneca and Sanofi. A. Spanevello received payment or honoraria as a speaker and a research grant from A. Menarini, AstraZeneca, Chiesi, GlaxoSmithKline, Merck Sharp and Dohme and Sanofi, and consultation fees from AstraZeneca, Chiesi, GlaxoSmithKline and Merck Sharp and Dohme. A. Vatrella received payment or honoraria as a speaker from A. Menarini, AstraZeneca, Boehringer Ingelheim, GlaxoSmithKline, Laboratori Guidotti, Lusofarmaco and Sanofi and a research grant from GlaxoSmithKline. C. Micheletto received payment or honoraria as a speaker from A. Menarini, AstraZeneca, Berlin Chemie, Boehringer Ingelheim, Chiesi, Firma, GlaxoSmithKline, Laboratori Guidotti, Novartis, Roche, Sanofi and Zambon and consultation fees from AstraZeneca, Chiesi and GlaxoSmithKline. S. Barbaglia, F. Bini, G. Pomponio, S. Tognella, and L. Zuccatosta declare no conflicts of interest.

## Supporting information

Supporting Information S1

Supporting Information S2

## Data Availability

The data that support the findings of this study are available from the corresponding author upon reasonable request.
