# Supplementary material for: Expert opinion on gray areas in asthma management: A lesson from the innovative project “revolution in asthma” of the Italian thoracic society (AIPO‐ITS)
Source: Clin Transl Allergy. 2025 Feb 9;15(2):e70037. doi: 10.1002/clt2.70037 (PMC11807766; doi:10.1002/clt2.70037)
Supplement: Supplementary file 1 — Supporting Information S1 [file CLT2-15-e70037-s002.docx]

**SUPPLEMENTARY MATERIAL**

**Supplementary Figure 1:** Summary of the project and the methodology used to identify the gray areas

**METHODS**

**Diagnosis**

The results of the Revolution project showed general adherence to LG recommendations [NICE, BTS/SIGN, NAEPP, EPR-3, GINA] regarding the need for objective examination to support the diagnosis of asthma; however, in real life, only 30% of the patients managed by the participating physicians were diagnosed using objective investigations.

The SC’s analysis of the results of the Revolution project revealed how the challenges in reaching a definite diagnosis depend not only on the accessibility of functional tests (approximately 50% of participants reported having spirometry and all reported having a PEF meter) but rather on the proper use of these tests in the context of the diagnostic investigation pathway. In fact, from a methodological point of view, no test can be considered a diagnostic gold standard. Therefore, diagnostic tests can increase the probability of asthma diagnosis but do not lead to a definitive diagnosis.^1–7^ Furthermore, the accuracy of tests depends on the pre-test probability of asthma of each patient, as outlined by Bayes’ theorem.^19^

Therefore, the diagnosis of asthma could be considered probabilistic and clinical-functional.^1–7^ This does not mean that examining the patient’s medical history is sufficient to make a diagnosis, but rather that it is necessary to carefully determine the pre-test probability of asthma through a structured survey that involves looking for the main features of asthma (e.g., daily or seasonal variability of symptoms, different respiratory symptoms associated to specific trigger factors)^20,21^ and associated risk factors (atopy, T2 inflammation, and rhino-sinus pathology).^21^

Once a significant (at least ≥50%) pre-test probability of asthma is established, the results of one or more diagnostic tests, chosen according to the care setting and the patient's characteristics/preferences (shared decision-making), will allow, in case of positivity, to determine the post-test probability of asthma (Bayes’ theorem)^19,22^ and to be sure of the diagnosis correctness. In line with the BTS/SIGN GLs, a definitive diagnosis is reached only after verification of the therapy's effectiveness with improvement and/or normalization of symptoms and respiratory function.^1^

The SC suggested a diagnostic algorithm, differentiated by care setting, that uses functional tests that are easy to implement in first-level outpatient clinics and allows a rapid diagnosis and an initial assessment of the patient's phenotype in second-level outpatient clinics (Supplementary Figure 1).

In first-level outpatient clinics, the SC suggested performing the bronchial reversibility test (BDR) on obstructed patients. If the test cannot be performed because the spirometry is normal or the test itself is too burdensome for the organizational level of the outpatient clinic, the SC suggested spirometry monitoring (FEV1) together with monitoring of symptoms between visits. A change in FEV1 values over 12% and greater than 200 ml in accordance with symptoms supports the diagnosis of asthma, with a higher probability associated with greater improvements.^1,23,24^

In first-level outpatient clinics, the SC suggested implementing longitudinal respiratory function surveys (FEV1 or PEF variability), as they are more likely to capture symptomatic states and give a positive result^20^ compared with tests such as the BDR, which is frequently negative in the outpatient setting.^25^ These tests also promote patient education and literacy and improve the dialogue with their GPs. Of note, these tests are characterized by good specificity but low sensitivity, so asthma cannot be ruled out in case of negativity.

Patients with negative results in the first-level setting but in whom the suspicion of asthma is well-founded, those who need a quick diagnosis, or complex patients with multimorbidity should be sent to second-level centers.

In second-level centers, the SC suggested performing the fractional exhaled nitric oxide measurement as the first test, followed by a BDR in the presence of an obstructive functional picture or a bronchial provocation test when spirometry is normal (tests characterized by high sensitivity). The fractional exhaled nitric oxide measurement can offer useful insights into the patient's phenotype and possible response to ICS^2,3,26–28^ and improve the accuracy of diagnosis.

**Control**

The results of the Revolution project showed that although there is a substantial formal agreement with guideline recommendations, a significant proportion of physicians do not use a structured survey approach, such as guided interviews and/or questionnaires and respiratory function tests to assess asthma control. The same is true for tools to improve patient education and literacy, empowerment, and adherence to therapy. This deficiency is not only due to a ''lack of time'' but also, as highlighted by some of the responses related to ''physicians' opinions”, from the mental reserves of physicians towards a change from a managerial role to shared management with patients (shared decision-making).

The main gray areas and needs identified by the SC refer to the necessity for implementable guidance on which tools to use (which should be as less time-consuming as possible) to assess control and improve adherence to therapy, patient education, and literacy and self-management skills.

The SC suggested conducting, both in the primary and secondary setting, a structured examination based on medical history assessment, possibly supported by objective tests, to improve the performance of clinical assessment of asthma control (Supplementary Figure 2). Follow-up visits should be performed at least once a year and tailored based on the severity of asthma and the level of control determined at the previous visit. Visits are also an opportunity to integrate educational interventions, improve patient-physician relationships, and assess exposure to risk factors and comorbidities.

**References**

1. The British Thoracic Society. BTS/SIGN British guideline on the management of asthma. 2019. https://www.brit-thoracic.org.uk/standards-of-care/guidelines/btssign-british-guideline-on-themanagement-of-asthma/. Accessed 28 Feb 2023.
2. National Institute for Health and Care Excellence (NICE) guideline NG80. Asthma: diagnosis, monitoring and chronic asthma management. 29 November 2017 – Last updated: 22 March 2021. <https://www.nice.org.uk/guidance/ng80>
3. NICE 2. Asthma: diagnosis, monitoring and chronic asthma management <https://www.nice.org.uk/guidance/ng80/evidence/a-increasing-ics-treatment-within-supported-selfmanagement-for-children-and-young-people-pdf-7079862638>
4. National Asthma Education and Prevention Program. Expert Panel Report 3 (EPR-3). Guidelines for the diagnosis and management of asthma – Summary report 2007. J Allergy Clin Immunol 2007;120:S94-S138.
5. Expert Panel Working Group of the National Heart, Lung, and Blood Institute (NHLBI) administered and coordinated National Asthma Education and Prevention Program Coordinating Committee (NAEPPCC); Cloutier MM, Baptist AP, Blake KV, et al. 2020 Focused Updates to the Asthma Management Guidelines: A Report from the National Asthma Education and Prevention Program Coordinating Committee Expert Panel Working Group. J Allergy Clin Immunol. 2020;146:1217-1270. doi: 10.1016/j.jaci.2020.10.003.
6. Global Initiative for Asthma (GINA) 2019. Global strategy for asthma management and prevention. 2019. <https://ginasthma.org/wp-content/uploads/2019/06/GINA-2019-main-report-June-2019-wms.pdf>
7. Global Initiative for Asthma (GINA) 2022. Global strategy for asthma management and prevention. 2022. https://ginasthma.org/gina-reports/ (Accessed 12 Jan 2023).
8. [www.revolutioninasma.it](http://www.revolutioninasma.it)
9. Gabbay J, le May A. Evidence based guidelines or collectively constructed "mindlines?" Ethnographic study of knowledge management in primary care. BMJ 2004;329:1013. doi: 10.1136/bmj.329.7473.1013.
10. Greenhalgh T, Howick J, Maskrey N; Evidence Based Medicine Renaissance Group. Evidence based medicine: a movement in crisis? BMJ 2014;348:g3725. doi: 10.1136/bmj.g3725.
11. King O, West E, Alston L, et al. Models and approaches for building knowledge translation capacity and capability in health services: a scoping review. Implement Sci 2024;19:7. doi: 10.1186/s13012-024-01336-0.
12. ERS Handbook <https://www.ersnet.org/science-and-research/development-programme/ers-clinical-practice-guidelines-statements-and-technical-standards/>
13. Nagavci B, Tonia T, Roche N, et al. European Respiratory Society clinical practice guidelines: methodological guidance. ERJ Open Res 2022;8:00655-2021. doi: 10.1183/23120541.00655-2021.
14. Plaza Moral V, Alobid I, Álvarez Rodríguez C, et al. GEMA 5.3. Spanish Guideline on the Management of Asthma. Open Respir Arch 2023;5:100277. doi: 10.1016/j.opresp.2023.100277.
15. Miles A, Loughlin M, Polychronis A. Medicine and evidence: knowledge and action in clinical practice. J Eval Clin Pract 2007;13:481-503. doi: 10.1111/j.1365-2753.2007.00923.x.
16. Tonelli MR. Integrating evidence into clinical practice: an alternative to evidence-based approaches. J Eval Clin Pract 2006;12:248-56. doi: 10.1111/j.1365-2753.2004.00551.x.
17. Greenhalgh T, Jackson C, Shaw S, Janamian T. Achieving research impact through co-creation in community-based health services: literature review and case study. Milbank Q 2016;94:392-429. doi: 10.1111/1468-0009.12197.
18. Jull J, Giles A, Graham ID. Community-based participatory research and integrated knowledge translation: advancing the co-creation of knowledge. Implement Sci 2017;12:150. doi: 10.1186/s13012-017-0696-3.
19. Hall GH. The clinical application of Bayes' theorem. Lancet 1967;2(7515):555-557. doi:10.1016/s0140-6736(67)90514-4.
20. Wang R, Murray CS, Fowler SJ, Simpson A, Durrington HJ. Asthma diagnosis: into the fourth dimension. Thorax 2021;76:624-631. doi:10.1136/thoraxjnl-2020-216421.
21. Tomita K, Sano H, Chiba Y, et al. A scoring algorithm for predicting the presence of adult asthma: a prospective derivation study. Prim Care Respir J 2013;22:51-8. doi: 10.4104/pcrj.2013.00005.
22. Sano H, Tomita K, Sano A, et al. Accuracy of objective tests for diagnosing adult asthma in symptomatic patients: A systematic literature review and hierarchical Bayesian latent-class meta-analysis. Allergol Int 2019;68:191-198. doi: 10.1016/j.alit.2018.08.013.
23. Global Initiative for Asthma (GINA) 2023. Global Strategy for Asthma Management and Prevention. Available at: <https://ginasthma.org/2023-gina-main-report/>.
24. Dean BW, Birnie EE, Whitmore GA, et al. Between-Visit Variability in FEV_1_ as a Diagnostic Test for Asthma in Adults. Ann Am Thorac Soc. 2018;15:1039-1046. doi: 10.1513/AnnalsATS.201803-211OC.
25. Appleton SL, Adams RJ, Wilson DH, Taylor AW, Ruffin RE; North West Adelaide Cohort Health Study Team. Spirometric criteria for asthma: adding further evidence to the debate. J Allergy Clin Immunol 2005;116:976-82. doi: 10.1016/j.jaci.2005.08.034.
26. Kuo CR, Spears M, Haughney J, et al. Scottish consensus statement on the role of FeNO in adult asthma. Respir Med 2019;155:54-57. doi: 10.1016/j.rmed.2019.07.010.
27. Taylor DR, Pijnenburg MW, Smith AD, De Jongste JC. Exhaled nitric oxide measurements: clinical application and interpretation. Thorax 2006;61:817-27. doi: 10.1136/thx.2005.056093.
28. American Thoracic Society; European Respiratory Society. ATS/ERS recommendations for standardized procedures for the online and offline measurement of exhaled lower respiratory nitric oxide and nasal nitric oxide, 2005. Am J Respir Crit Care Med 2005;171:912-30. doi: 10.1164/rccm.200406-710ST.
