# Supplementary material for: Expert opinion on gray areas in asthma management: A lesson from the innovative project “revolution in asthma” of the Italian thoracic society (AIPO‐ITS)
Source: Clin Transl Allergy. 2025 Feb 9;15(2):e70037. doi: 10.1002/clt2.70037 (PMC11807766; doi:10.1002/clt2.70037)
Supplement: Supplementary file 2 — Supporting Information S2 [file CLT2-15-e70037-s001.pdf]

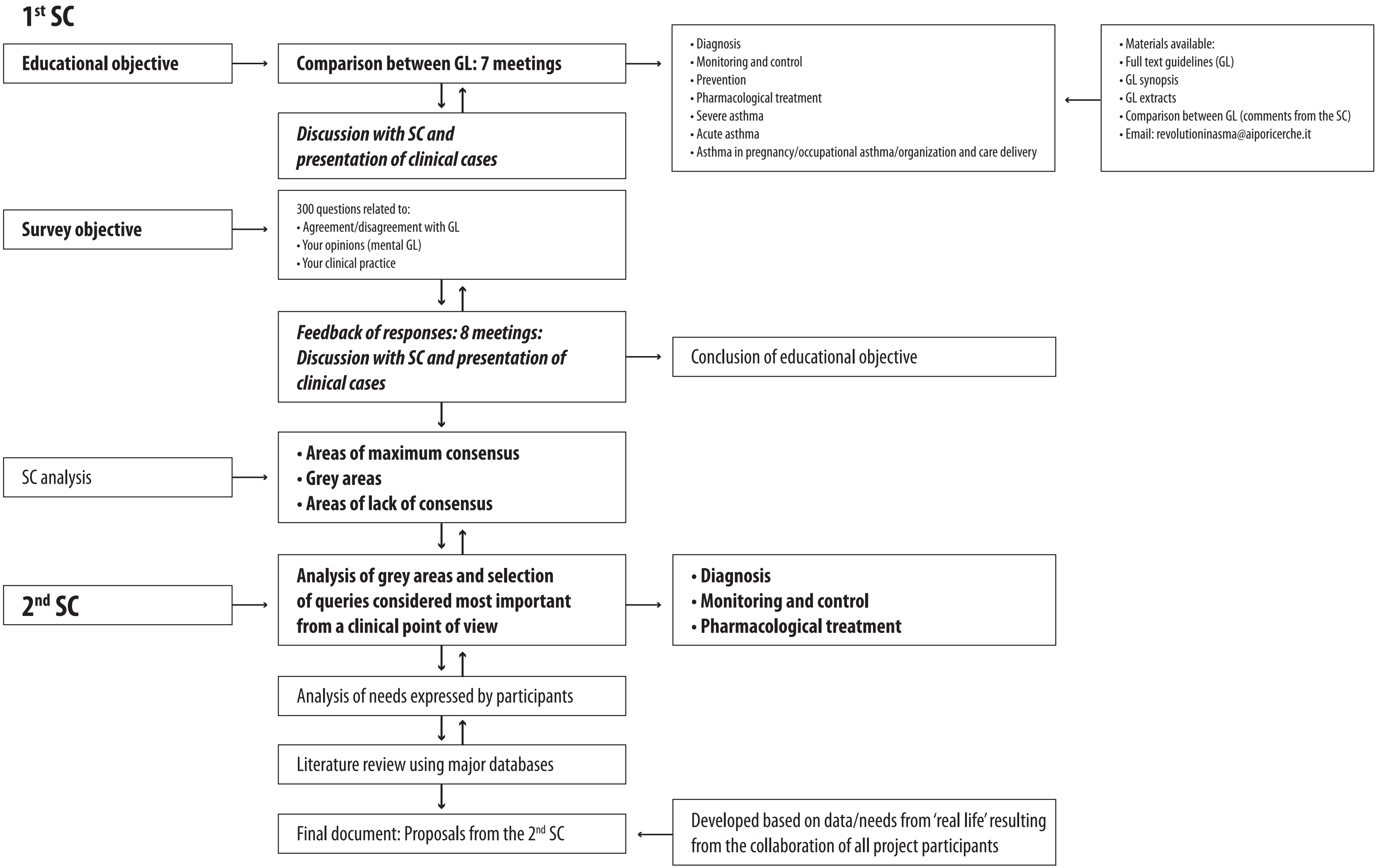
1st SC
Educational objective
Comparison between GL: 7 meetings
• Diagnosis
• Monitoring and control
• Prevention
• Pharmacological treatment
• Severe asthma
• Acute asthma
• Asthma in pregnancy/occupational asthma/organization and care delivery
• Materials available:
• Full text guidelines (GL)
• GL synopsis
• GL extracts
• Comparison between GL (comments from the SC)
• Email: revolutioninasma@aiporicerche.it
Discussion with SC and presentation of clinical cases
Survey objective
300 questions related to:
• Agreement/disagreement with GL
• Your opinions (mental GL)
• Your clinical practice
Feedback of responses: 8 meetings: Discussion with SC and presentation of clinical cases
Conclusion of educational objective
SC analysis
• Areas of maximum consensus
• Grey areas
• Areas of lack of consensus
2nd SC
Analysis of grey areas and selection of queries considered most important from a clinical point of view
• Diagnosis
• Monitoring and control
• Pharmacological treatment
Analysis of needs expressed by participants
Literature review using major databases
Final document: Proposals from the 2nd SC
Developed based on data/needs from 'real life' resulting from the collaboration of all project participants
